# Pathology of Hysteria

**Published:** 1858-01

**Authors:** N. B. Drewry

**Affiliations:** Sharon Grove, Georgia


### ARTICLE III.

*Pathology of Hysteria.* By N. B. Drewry, M. D., of Sharon Grove, Georgia.

Much has been said by those high in authority, within the last century, in regard to the Pathology of Hysteria; and much too, to no purpose—as the subject, even at the present day is far from being settled.

May we have Hysteria in the male, is a question always freely discussed by those who have attempted a solution of the Pathology of this protian disease. Without any intention of entering the list of those who have engaged in the discussion of the vexed question we propose here only a brief report of two cases, which recently came under our observation, and which support the opinions of those who contend that we may have Hysteria in the male.

Mr. J——, a farmer aged 65 years, short, thick body, large neck and head, scorbutic diathesis, wishing to extend his farm, turned the public highway, which gave offence to his neighbors, and resulted in an appeal to law. Upon being informed of the course his neighbors had adopted, he was stricken down with convulsions, and by his family was supposed to be dying. I was immediately sent for in great haste, and upon my arrival found the convulsions passing off, and he able to talk.

Complained of pain in the nape of the neck, dizziness, distention of the Stomach and Bowels with a sensation as if something passed from the stomach to the throat, and then receded. Uneasiness of the spine with indisposition to keep his bed. Pulse 45 or 50 per minute. He was relieved to a very great extent by Caustic irritation to the spine, Valerian and Quinine with an occasional blue pill.

Soon after this, Mr. J—— learned, that he had gained a suit, in which, there was a considerable amount of property involved, and upon the reception of the intelligence evinced unusual hilarity.

Upon receiving notice of an appeal and a continuation of the suit, he had a second attack, which was more severe than the first. Even now, upon the reception of unpleasant intelligence, he is immediately attacked with this hysterical form of disease.

In October, 1855, I was called to see a gentleman with spare form and delicate constitution. Used tobacco and alcoholic drinks to a considerable extent, the latter, however, not to the extent to produce a decided impression upon the system.—

Found him with great pain in the region of the Stomach, bowels distended. Complained of a peculiar sensation about the throat, as in the above case, which he described as something rising up from the Stomach to the Throat, which nearly suffocated him—a kind of choking. Some uneasiness about the spine—pulse natural—tongue red and fevered. Upon going to sleep is suddenly aroused with muscular contraction, suffocation, &c.

He was relieved by a Cathartic of Calomel and Jalap ; opiates increased the convulsive action. I ordered tonics, farinaceous diet, and alkaline drinks ; forbid the use of tobacco and alcoholic drinks, and his recovery was speedy. The cause of the nervous symptoms was evidently a form of indigestion.

Sometime after this first attack, he resumed the use of tobacco and stimulants, when a partial relapse ensued. To the above, I might add several other similar cases, but as they do not differ in any essential point from the two just related, we have thought it unnecessary to occupy time and space with the detail. In the above two cases, we had the symptoms most prominent in hysterical females. But, shall we call it hysteria ? or should we rather call such afflictions, indigestion, diseased Liver, Stomach, Bowels, &c. &c., with a train of nervous or hysterical symptoms ?

